# N1-Methylpseudouridine-Modified mRNA-Transfected Dendritic Cells Enable Efficient Generation of Functional Tumor Antigen-Specific Cytotoxic T Lymphocytes

**DOI:** 10.3390/ijms27209191

**Published:** 2026-10-16

**Authors:** Tiffany Chor Kiu Ngan, Mouldy Sioud

**Affiliations:** Department of Cancer Immunology, Institute for Cancer Research, Oslo University Hospital, Ullernchausseen 70, 0379 Oslo, Norway

**Keywords:** dendritic cells, modified mRNA, cytotoxic T cells, mRNA vaccines, tumor-associated antigens

## Abstract

Efficient generation of tumor-specific cytotoxic T lymphocytes (CTLs) remains challenging. Although mRNA-based approaches enable the delivery and expression of diverse tumor-specific antigens, unmodified mRNA can trigger innate immune responses that suppress translation. Here, we investigated whether 1-methyl-pseudouridine (m^1^Ψ)-modified mRNA enhances antigen expression in human dendritic cells (DCs) and promotes the induction of antigen-specific CTLs, using survivin and MART-1 as model tumor-associated antigens. Remarkably, m^1^Ψ modification increased protein production in DCs by up to 20-fold compared with unmodified mRNA. DCs transfected with m^1^Ψ-modified mRNAs efficiently primed survivin- and MART-1-specific CD8^+^ T cells from autologous peripheral blood mononuclear cells of healthy donors, as demonstrated by HLA class I peptide–MHC dextramer staining. Expanded CTL lines exhibited potent effector functions, including IFN-γ production, CD107a mobilization, and robust cytotoxicity against peptide-pulsed T2 cells and tumor cell lines expressing survivin or MART-1. Translation of the engineered mRNA variants in human HeLa cells and DCs is strongly dependent on the poly(A) tail. Together, these findings demonstrate that m^1^Ψ modification substantially enhances antigen expression and supports the efficient generation of functional, antigen-specific CTLs, providing a promising platform for next-generation mRNA-based cancer vaccines and adoptive T-cell therapies.

## 1. Introduction

Conventional cancer treatments, including chemotherapy, radiotherapy, and surgery, remain central to cancer management but are frequently limited by toxicity and damage to healthy tissues. Cancer immunotherapy offers a fundamentally different therapeutic paradigm by harnessing the immune system to selectively recognize and eliminate malignant cells, either by enhancing antitumor immune responses or by overcoming tumor-mediated immunosuppression [1,2,3]. Among the various immunotherapeutic strategies, therapeutic cancer vaccination has emerged as a promising approach for inducing tumor-specific T-cell responses [4,5]. Messenger RNA (mRNA)-based vaccines have attracted considerable interest because of their favorable safety profile, versatility, and potential for rapid and scalable manufacturing [6]. In vitro-transcribed (IVT) mRNA enables transient expression of tumor antigens without the risk of genomic integration, making it an attractive platform for cancer vaccination [7,8,9]. However, early development of mRNA therapeutics was constrained by poor stability, inefficient translation, and potent activation of innate immune pathways [7]. Exogenous RNA is sensed by multiple innate immune receptors, including endosomal Toll-like receptors (TLRs; TLR3, TLR7, and TLR8) and cytosolic RIG-I-like receptors such as RIG-I and MDA5 [10]. Activation of these pathways induces antiviral responses characterized by the production of type I interferons and a large panel of pro-inflammatory cytokines [10,11,12,13]. Although the recognition of exogenous RNAs by innate immunity is essential for host defense against viral infection and can contribute to the activation of adaptive immunity, excessive sensing of therapeutic mRNA can be detrimental by inhibiting translation of the encoded protein/antigen. This is of particular importance for repeat dosing of RNA therapeutic drugs such as small interfering RNAs (siRNAs).

A major advance in the development of RNA therapeutics, including mRNA and siRNA, was the discovery that incorporation of modified nucleosides can substantially reduce innate immune recognition. In this regard, we and others have demonstrated that naturally occurring or chemically modified nucleosides, including 2′-*O*-methyl nucleosides, pseudouridine (Ψ), and N1-methylpseudouridine (m1Ψ), can attenuate the activation of innate immune sensors and reduce inflammatory cytokine responses [14,15,16,17]. Notably, 2′-*O*-methyl-modified RNA not only suppresses TLR signaling but also acts as a TLR7/8 antagonist, thereby blocking the recognition of both self- and non-self-immunostimulatory RNAs [18]. In the context of mRNA therapeutics, substitution of uridine with Ψ or m1Ψ markedly enhances mRNA translational capacity while improving tolerability [19,20]. These findings have been instrumental in the development and clinical success of the two approved COVID-19 mRNA vaccines, establishing nucleoside modification as a transformative strategy for mRNA-based therapeutics [21,22,23,24].

Given their important role in linking innate and adaptive immunity, as well as in antigen presentation and cross-presentation, dendritic cells (DCs) have been extensively investigated as cellular cancer vaccines [25]. These approaches typically involve the ex vivo differentiation of autologous monocytes into DCs, loading with tumor antigens, and subsequent reinfusion into patients [7]. Although such strategies can induce measurable antigen-specific immune responses, their clinical efficacy has generally remained limited, suggesting that the magnitude and quality of the induced T-cell response may be insufficient to overcome the immunological barriers imposed by established tumors [26]. One major challenge is that tumor-associated antigens (TAAs) are self-proteins and are therefore subject to both central and peripheral immune tolerance [27,28]. Consequently, effective cancer vaccination must elicit T-cell responses that are sufficiently strong to overcome pre-existing tolerance while maintaining appropriate antigen specificity [29,30]. Increasing the abundance of TAA-derived peptides available for MHC class I presentation represents one potential strategy to achieve this goal. Enhanced intracellular antigen expression could increase both the quantity and diversity of antigen-derived peptide–MHC class I complexes displayed by DCs, thereby increasing the likelihood of activating low-frequency tumor-reactive T cells that have escaped immune tolerance. Indeed, analogous mechanisms have been implicated in autoimmune responses, in which autoreactive T cells can become activated and mediate tissue destruction under conditions of enhanced or dysregulated antigen presentation [31,32,33]. These considerations highlight the importance of optimizing mRNA design not only to minimize unwanted innate immune activation but also to maximize antigen production within DCs. Although nucleoside-modified mRNAs have been extensively investigated in a variety of experimental and clinical settings [34], direct comparisons of nucleoside-modified and unmodified mRNA with respect to antigen expression and subsequent induction of tumor-specific cytotoxic T lymphocytes (CTLs) in human DCs remain limited. Whether the enhanced antigen expression achieved through m^1^Ψ modification, together with m^1^Ψ-mediated ribosome stalling, translates into more effective priming of functional tumor-specific CD8^+^ T cells therefore remains an important unanswered question.

In this study, we investigated the impact of m^1^Ψ modification on antigen expression and T-cell priming by human DCs using survivin and MART1 as model TAAs. Both antigens represent clinically relevant targets for cancer immunotherapy [35]. Survivin, a member of the inhibitor of apoptosis protein family, is highly expressed across a broad range of human malignancies but is absent or expressed at very low levels in most normal adult tissues [36]. Elevated survivin expression has been associated with tumor progression, resistance to chemotherapy, and poor clinical outcomes, further supporting its potential as a therapeutic target [37]. MART1 (Melanoma Antigen Recognized by T cells 1), also known as Melan-A, is a melanocyte differentiation antigen expressed in the majority of melanomas and is one of the best-characterized targets for melanoma-directed T-cell immunotherapy [38]. Here, we demonstrated that DCs transfected with m^1^Ψ-modified mRNA can exhibit substantially enhanced antigen expression and efficient priming of survivin- and MART1-specific CD8^+^ T cells from autologous peripheral blood mononuclear cells (PBMCs) obtained from healthy donors. The resulting CTLs recognized endogenously processed survivin- or MART1-derived epitopes presented in the context of HLA-A*02:01 (HLA-A2) and showed potent effector functions, including cytokine production, degranulation, and tumor-cell killing.

## 2. Results

### 2.1. m^1^Ψ-Modified Survivin mRNA Enhances Antigen Expression in Dendritic Cells

**Figure 1:**
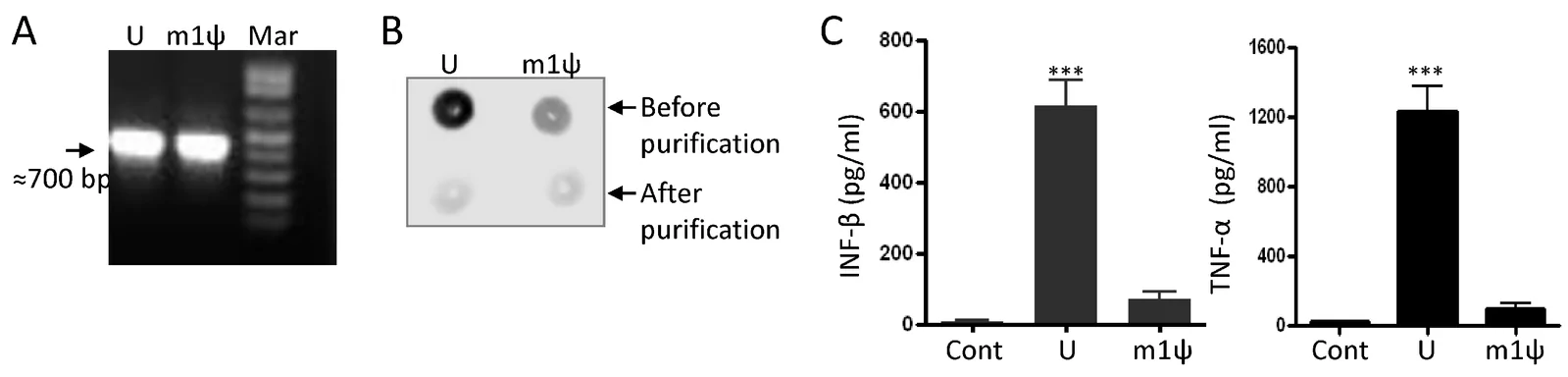
Characterization and innate immune sensing of survivin IVT mRNA. (**A**) Integrity of unmodified and m^1^Ψ-modified survivin mRNA. Following cellulose-based purification, 100 ng of each mRNA preparation was analyzed by electrophoresis on a 1.2% agarose gel. Both transcripts displayed a predominant band at the expected size. (**B**) Detection of dsRNA by-products in IVT mRNA. dsRNA content was assessed in unpurified (before) and cellulose-purified (after) mRNA preparations by dot blot using the dsRNA-specific J2 monoclonal antibody [39]. For each sample, 500 ng of mRNA was applied to the membrane. (**C**) Innate immune sensing of survivin mRNA by human monocyte-derived immature DCs (iDCs). iDCs were seeded in 48-well plates (2 × 10^5^/well/600 μL) and transfected with either 500 ng dose of purified uridine mRNA (U) or m^1^Ψ-modified survivin mRNA (m^1^Ψ). After 18 h, TNF-α and IFN-β concentrations in culture supernatants were measured by ELISA. Values are presented as mean ± SD of three independent experiments, each performed in duplicate, using iDCs from different donors. *** *p* < 0.001.

Most comparative studies of m^1^Ψ-modified and unmodified mRNA have been performed in non-immune cell lines, such as HEK293T cells, whereas their performance in clinically relevant antigen-presenting cells, particularly DCs, has been limited. Because DCs are central to the initiation of antitumor T-cell responses, we first investigated whether m^1^Ψ modification could improve antigen expression in human DCs. For the first experiments, we selected survivin, a broadly expressed TAA. Unmodified and m^1^Ψ-modified IVT mRNAs were synthesized under identical transcription conditions and subsequently purified using cellulose-based purification to remove dsRNA contaminants. The integrity of the resulting transcripts was first assessed by agarose gel electrophoresis. Both unmodified and m^1^Ψ-modified mRNAs displayed a predominant band at the expected molecular size, with no evidence of degradation, confirming the high integrity of the purified transcripts (Figure 1A). We next assessed dsRNA by-products in crude transcription reactions using the J2 dsRNA-specific antibody [39]. A substantially stronger J2 signal was detected in reactions containing uridine compared with those containing m^1^Ψ, indicating that m^1^Ψ incorporation inhibited the formation of dsRNA species during IVT, as exemplified in Figure 1B. Following cellulose purification, J2 reactivity was markedly reduced and comparable between the two mRNA preparations, confirming efficient removal of dsRNA contaminants by the purification procedure.

We next examined whether differences in nucleoside composition affected the innate immune response to the purified mRNAs in human immature DCs (iDCs). These cells express multiple RNA-sensing pathways, including PKR, RIG-I, and endosomal TLR3, TLR7, and TLR8 [11]. Despite efficient removal of dsRNA, transfection with uridine-containing mRNA induced substantial production of IFN-β and TNF-α, whereas m^1^Ψ-modified mRNA elicited minimal cytokine production (Figure 1C, *p* < 0.001). Previous studies have shown that uridine-containing single-stranded RNAs are detected by endosomal TLR7 and TLR8 [10]. The same pattern was reproducibly observed across five independent IVT mRNA preparations, demonstrating that the reduced innate immune activation associated with m1Ψ modification was highly consistent.

**Figure 2:**
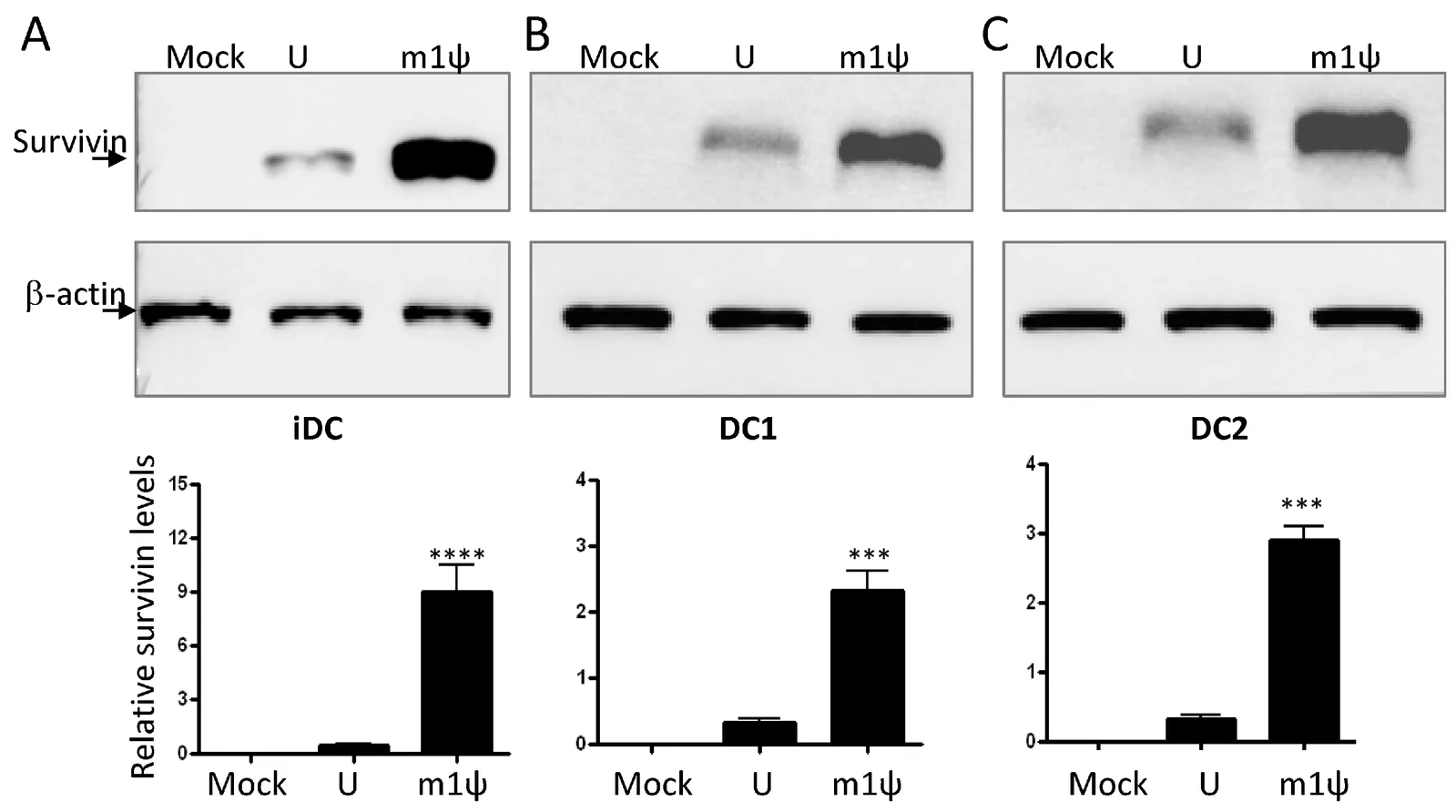
m^1^Ψ modification enhances survivin expression in human dendritic cells. (**A**) Monocyte-derived immature DCs (iDC) were transfected with 500 ng dose of unmodified (U) or m^1^Ψ-modified survivin mRNA (m^1^Ψ). After 18 h, survivin protein expression was analyzed by Western blotting using a survivin-specific monoclonal antibody. Survivin expression was quantified by densitometry and normalized to β-actin for each sample (**lower** panel, *n* = 3). (**B**,**C**) Survivin expression in circulating conventional dendritic cell subsets, cDC1and cDC2 (n = 3). The cells were purified from peripheral blood using magnetic bead-conjugated monoclonal antibodies and transfected with 500 ng dose of either unmodified (U) or m^1^Ψ-modified survivin mRNA (m^1^ψ). After 18 h, survivin protein expression was analyzed by Western blotting. Survivin expression was quantified by densitometry and normalized to β-actin for each sample (**lower** panels). Values are presented as mean ± SD of two-three independent experiments. *** *p* < 0.001, **** *p* < 0.0001.

Having established a marked difference in innate immune activation, we next asked whether m^1^Ψ modification could improve the translation of mRNA in human iDCs. Cells were transfected with equivalent amounts of lipid-formulated survivin mRNA containing either uridine or m^1^Ψ. Whereas survivin expression following transfection with uridine mRNA was minimal or frequently undetectable, m^1^Ψ-modified mRNA induced robust expression of survivin protein (Figure 2A). Quantitative analysis across three independent experiments demonstrated an approximately 20-fold increase in survivin expression following m^1^Ψ modification compared with uridine mRNA (Figure 2A, lower panel, *p* < 0.0001). At the mRNA doses tested (0.5–1.5 μg/transfection), survivin expression from uridine mRNA remained consistently low, whereas m^1^Ψ-modified mRNA produced readily detectable and substantially higher protein levels.

To determine whether circulating conventional dendritic cells (cDCs) exhibit a similar response to mRNA as monocyte-derived iDCs, we isolated the two major circulating cDC subsets, cDC1 (CD141/BDCA-3^+^) and cDC2 (CD1c/BDCA-1^+^), from peripheral blood mononuclear cells (PBMCs). cDCs are specialized antigen-presenting cells with a central role in initiating and shaping adaptive immune responses, including the priming of naïve T cells [40]. We therefore assessed survivin expression following transfection with either uridine or m^1^Ψ-modified survivin mRNA. Incorporation of m^1^Ψ enhanced survivin protein expression by approximately 6–10-fold in both cDC1 and cDC2 compared with uridine-containing mRNA, which resulted in only low protein expression (Figure 2B,C, upper and lower panels, *p* < 0.001). These data indicate that the translational advantage conferred by m^1^Ψ modification extends beyond monocyte-derived iDCs to circulating human cDC subsets, highlighting the potential of m^1^Ψ-modified mRNA for efficient antigen delivery to physiologically relevant DC populations.

### 2.2. m^1^Ψ-Modified mRNA-Transfected DCs Enhance the Expansion of Antigen-Specific CD8^+^ T Cells

**Figure 3:**
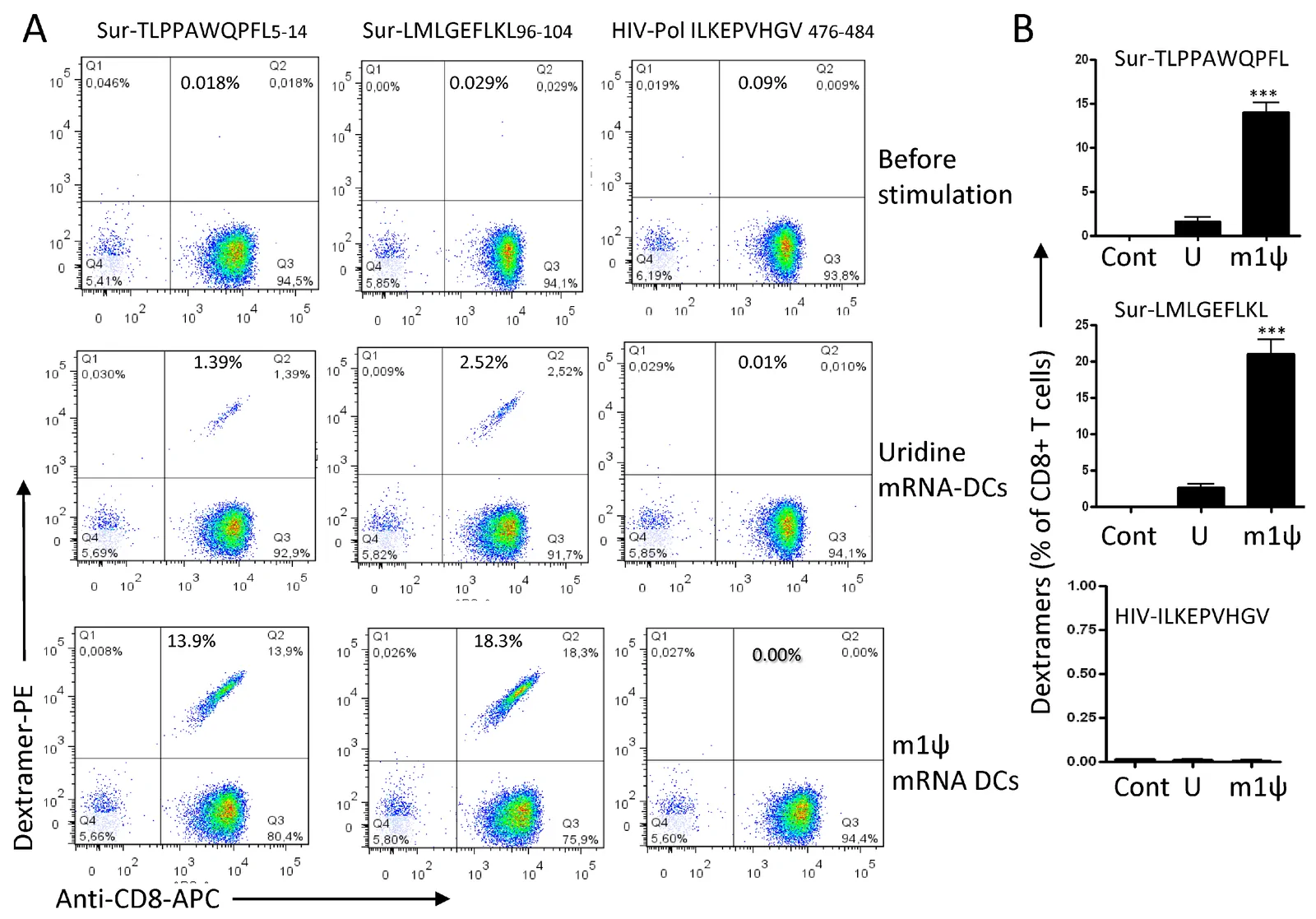
Efficient expansion of survivin-specific CTLs using autologous DCs transfected with m1Ψ-modified survivin mRNA. (**A**) Immature DCs (iDCs) generated from HLA-A2^+^ healthy donors were transfected with either uridine-containing survivin mRNA (U) or m^1^Ψ-modified survivin mRNA (m^1^Ψ), matured, and used to stimulate autologous T cells. T cells were co-cultured with irradiated autologous DCs for repeated stimulation. On day 28, survivin-specific CTLs were assessed by flow cytometry using PE-labeled HLA-A2 dextramers loaded with the survivin-derived peptides TLPPAWQPFL_5–14_ or LMLGEFLKL_96–104_ as indicated on the top of the panels. An HLA-A2-restricted HIV-1 Pol peptide (ILKEPVHGV_476–484_) was included as an irrelevant control. APC-labeled anti-CD8 antibody was used to identify CD8^+^ T cells. Cells were analyzed by flow cytometry and gated on CD8^+^ lymphocytes; the percentages of cells in each quadrant are indicated. Before stimulation (day 0), survivin–dextramer-positive CD8^+^ T cells were detected at frequencies close to background levels. (**B**) Quantification of survivin/dextramer-positive CD8^+^ T cells from three independent experiments, each performed in duplicate, using CTLs derived from different healthy donors. Data are presented as the mean the percentage of dextramer-positive cells among CD8^+^ T cells ± SD. *** *p* < 0.002.

CTLs play a central role in tumor cell elimination, and their efficient induction is a critical objective of therapeutic cancer vaccination [41]. We therefore investigated whether the enhanced antigen expression achieved with m^1^Ψ-modified survivin mRNA translated into more efficient expansion of survivin-specific CD8^+^ T cells. Autologous T cells from healthy HLA-A2^+^ donors were repeatedly stimulated with mature DCs transfected with either uridine or m^1^Ψ-modified survivin mRNA (500 ng dose). Following four rounds of stimulation at 7-day intervals, the resulting polyclonal CTL populations were analyzed using HLA-A2 peptide–MHC dextramers specific for two well-characterized survivin-derived epitopes, TLPPAWQPFL (residues 5–14) and LMLGEFLKL (residues 96–104, anchor-modified immunodominant epitope) [42,43]. An HLA-A2-restricted HIV-1 Pol epitope, ILKEPVHGV (residues 476–484), was included as a negative control. DCs transfected with m^1^Ψ-modified survivin mRNA induced substantially higher frequencies of survivin-specific CD8^+^ T cells than DCs transfected with uridine mRNA (Figure 3A, representative example). Quantitative analysis demonstrated a significant enrichment of CD8^+^ T cells recognizing both the TLPPAWQPFL_5–14_ and LTLGEFLKL_96–104_ epitopes following stimulation with m1Ψ-transfected DCs (Figure 3B). Frequencies of dextramer-positive cells reached 13% ± 3.5% and 20% ± 6%, respectively, compared with 1.5% ± 1% and 2.5% ± 0.5%, respectively, in cultures stimulated with uridine mRNA-transfected DCs (*p* < 0.002). Importantly, the two distinct survivin-specific dextramers demonstrate that m^1^Ψ-modified mRNA-transfected DCs could expand T-cell populations directed against multiple survivin epitopes rather than a single dominant specificity. Collectively, these data indicate that the enhanced antigen expression conferred by m^1^Ψ modification translates into substantially more efficient expansion of polyclonal, multi-epitope survivin-specific CD8^+^ T-cell responses.

**Figure 4:**
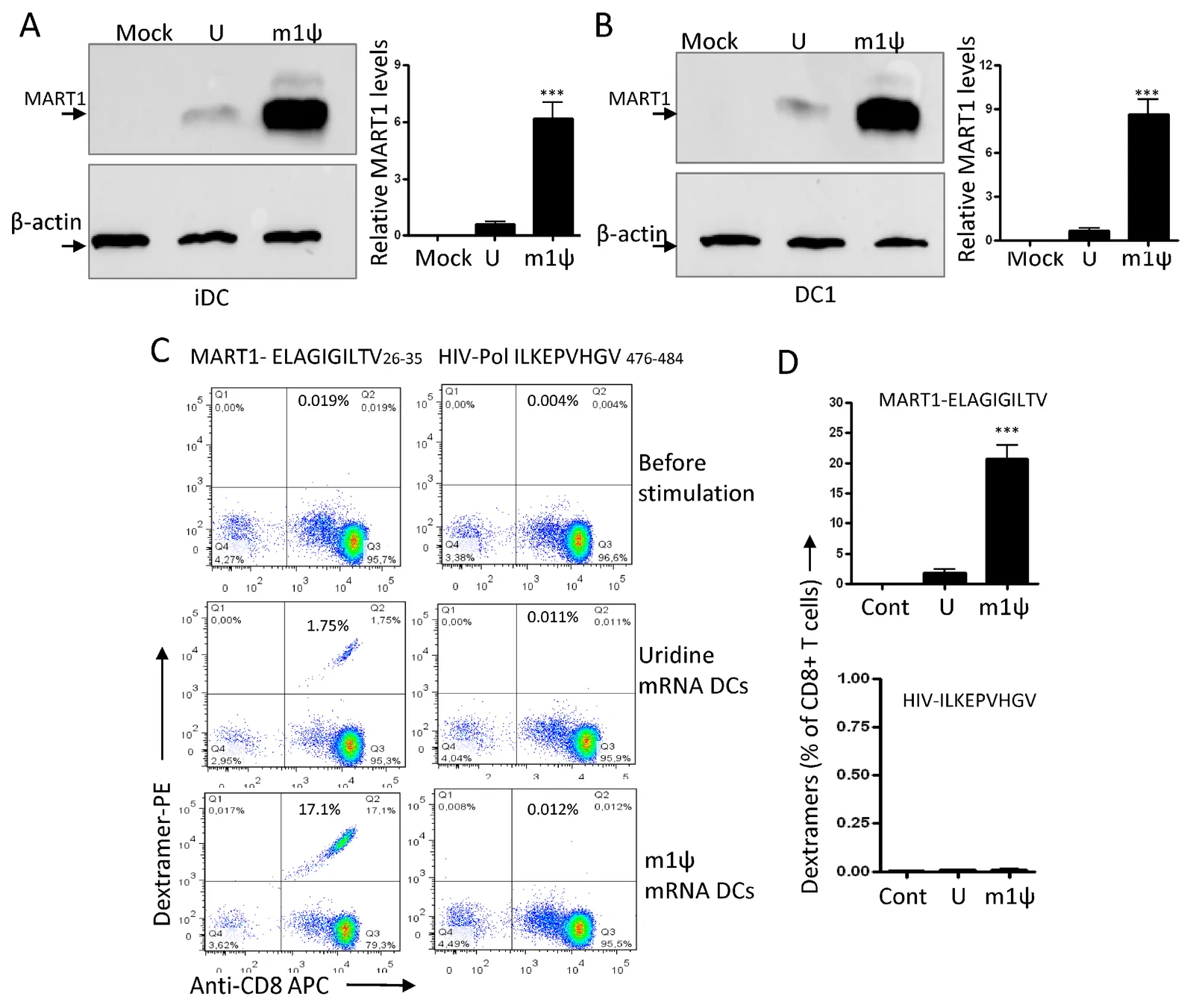
m1Ψ-modified MART-1 mRNA enhances antigen expression and promotes MART-1-specific CTL expansion. (**A**) Monocyte-derived immature DCs (iDCs) were transfected with 500 ng of either uridine (U-) or m1Ψ-modified MART-1 mRNA (m1Ψ). After 18 h, MART-1 protein expression was analyzed by Western blotting using a MART-1-specific monoclonal antibody. MART-1 expression was quantified by densitometry and normalized to β-actin for each sample (right panel; n = 3). (**B**) MART-1 expression in circulating conventional dendritic cells (cDC1). Cells were isolated from peripheral blood of healthy donors, transfected with 500 ng of either uridine (U) or m^1^Ψ-modified MART1 mRNA (m^1^ψ), and MART1 expression was analyzed by Western blotting. The signals were quantified by densitometry and normalized to β-actin for each sample (right panel; n = 3). (**C**) Generation of MART1-specific CTLs. Monocyte-derived iDCs from HLA-A2^+^ healthy donors were transfected with either uridine-containing MART1 mRNA or m1Ψ-modified MART-1 mRNA, matured, and used to stimulate autologous CD8^+^ T cells. T cells were repeatedly stimulated with irradiated DCs. On day 26, MART-1-specific CTLs were assessed by flow cytometry using PE-labeled HLA-A2 dextramers loaded with the MART-1-derived peptide ELAGIGILTV_26–35_. An HLA-A2-restricted HIV-1 Pol peptide (ILKEPVHGV_476–484_) was included as an irrelevant control. APC-labeled anti-CD8 antibody was used to identify CD8^+^ T cells. Cells were gated on CD8^+^ lymphocytes. The percentages of cells in each quadrant are indicated. (**D**) Graphs represent data from CTLs generated in three independent experiments, each performed in duplicate, using CTLs derived from different healthy donors. Data are presented as mean ± SD. *** *p* < 0.002.

In the next set of experiments, we investigated whether the data obtained with survivin mRNA could also be extended to other TAAs. For this purpose, we selected the melanoma antigen recognized by T cells 1, also known as Melan-A (MART1). MART1 is highly expressed in many melanoma tumors [44]. Like survivin, the translatability of m^1^Ψ-modified MART1 mRNA was approximately 10 and 18-fold higher in iDC and DC1, respectively, compared with uridine mRNA (Figure 4A,B, *p* < 0.001). After 4 cycles of stimulation, a significant fraction of the m^1^Ψ MART-1 mRNA transfected DCs induced CTLs stained with the MART1 dextramer (ELAGIGILTV, residues 26–35 anchor-modified epitope) compared to CTLs generated with DC-transfected with uridine mRNA (Figure 4C, as a representative example). Data from three independent experiments are shown in Figure 4D. Approximately 17.2% ± 6.5% of the CTLs generated with DC-transfected with m^1^ψ mRNA stained with MART1-dextramer compared to those generated with DC-transfected with uridine mRNA (2.5% ± 0.62%, *p* < 0.002).

### 2.3. Functional Competence of CTLs Generated with m1Ψ-Modified mRNA

**Figure 5:**
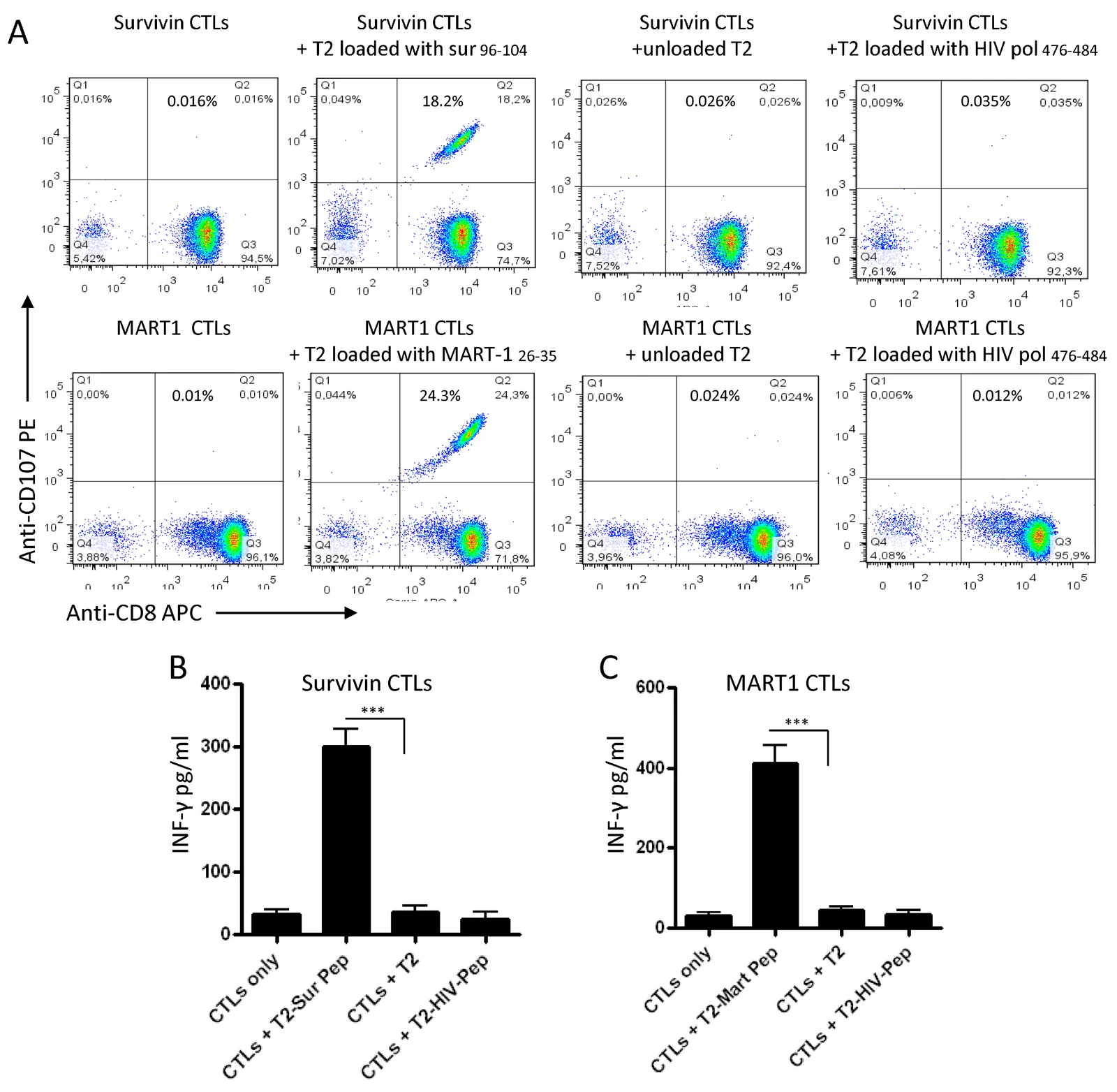
Functional activity of tumor antigen-specific CTLs. (**A**) As indicated T2 cells were loaded with either the survivin-derived peptide LTLGEFLKL_96–104_ or control HIV-1 Pol peptide ILKEPVHGV_476–484_ and co-cultured with CTLs (**upper** panels). Unloaded T2 cells were included as a negative control. Similarly, T2 cells were loaded with MART1 peptide_2__6–35_ or HIV control peptide (**lower** panels). Following incubation for 6 h at 37 °C, CTL degranulation was assessed by measuring surface CD107a mobilization by flow cytometry. Cells were gated on CD8^+^ lymphocytes. (**B**,**C**) Assessment of cytokine production, as in (**A**), CTLs were co-cultured with peptide-loaded or control T2 cells for 16 h. IFN-γ concentrations in culture supernatants were measured by ELISA. Error bars represent SD of three independent experiments, each performed in triplicate, using CTLs derived from different healthy donors. *** *p* < 0.001.

The ability to generate tumor antigen-specific T cells is only therapeutically meaningful if these cells acquire the functional capacity to recognize and eliminate target cells. We therefore assessed the functional competence of CTLs generated using DCs transfected with m^1^Ψ-modified survivin or MART1 mRNA. CTL activation was evaluated by measuring degranulation and IFN-γ production following antigen-specific stimulation. Degranulation was assessed by surface mobilization of lysosomal-associated membrane protein 1 (LAMP-1/CD107a), which reflects the release of cytotoxic granules from activated CTLs [45]. As shown in representative experiments (Figure 5A), stimulation with T2 cells loaded with either the wild-type survivin LTLGEFLKL_96–104_ or MART1 EAAGIGILTV_26–35_ peptide induced robust CD107a mobilization (second panels). In contrast, no significant degranulation was observed in response to unloaded T2 cells or T2 cells loaded with control HIV-1 Pol peptide ILKEPVHGV_476–484_ (two last panels). Consistent with the degranulation response, CTLs produced substantially higher levels of IFN-γ following stimulation with the corresponding tumor antigen-derived peptides compared with the irrelevant control peptide (Figure 5B,C, *p* < 0.001). These data demonstrate that the dextramer-positive CTLs generated with m^1^Ψ-modified mRNA-transfected autologous DCs were functionally responsive to their cognate tumor antigen-derived peptides.

**Figure 6:**
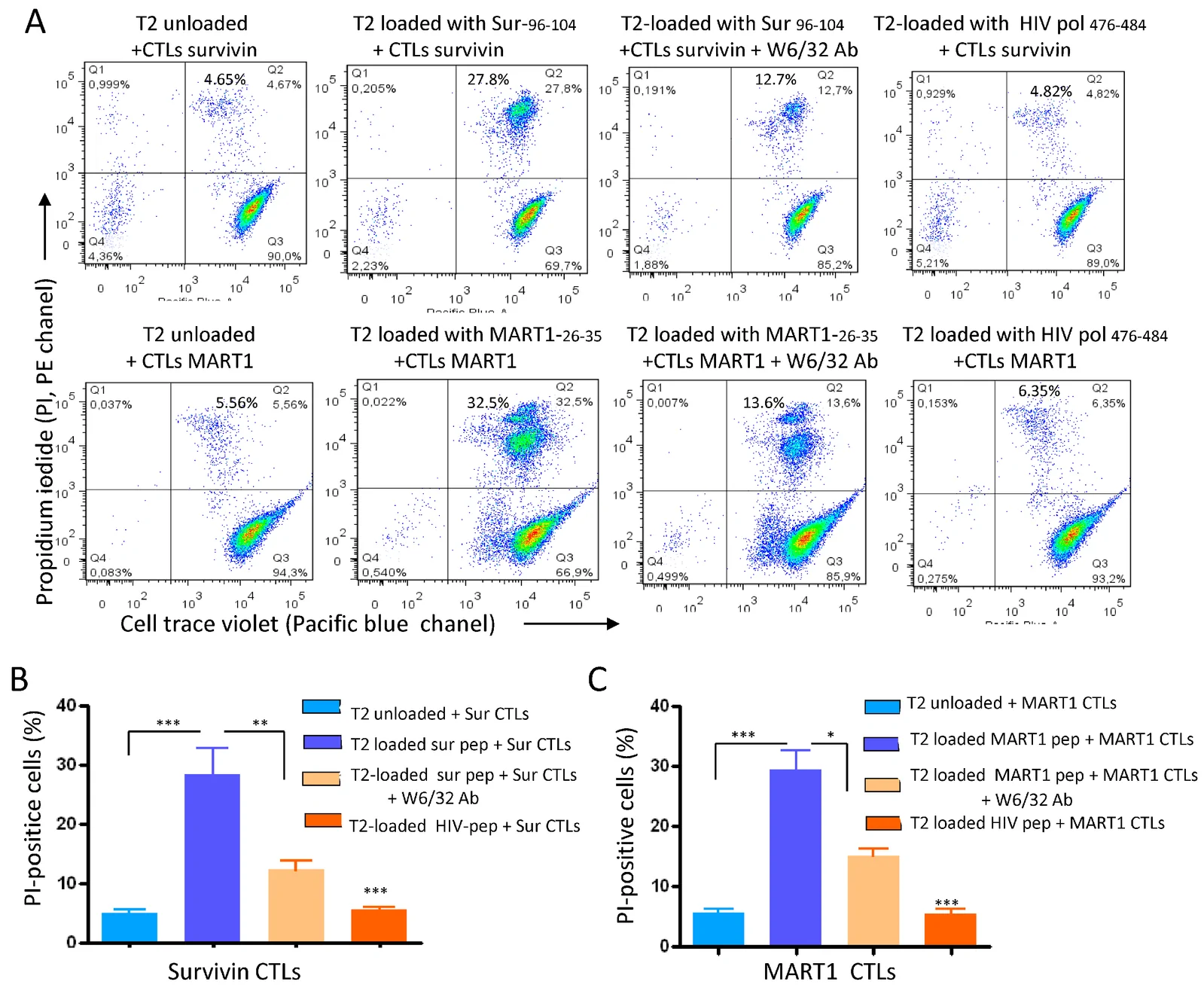
Antigen-specific cytotoxicity of CTLs generated with m^1^Ψ-modified mRNA-transfected DCs. (**A**) Cytotoxic activity of CTLs was assessed using a flow cytometry-based assay. Target T2 cells were labeled with CellTrace Violet and loaded with either the survivin-derived peptide LTLGEFLKL_96–104_ (**upper** panels) or MART1-derived peptide EAAGIGILTV_26–35_ (**Lower** panels). As a control, the cells were loaded with HIV-1 Pol peptide ILKEPVHGV_476–484_. The cells were also pre-incubated with W6/32 MHC-I blocking antibody prior to loading with survivin or MART1 peptides. Peptide-loaded target cells were co-cultured with the corresponding CTLs at an effector-to-target ratio of 10:1 for 6 h at 37 °C. Following co-culture, cells were stained with propidium iodide (PI) to distinguish viable from dead target cells and analyzed by flow cytometry. Representative dot plots are shown for survivin-specific CTLs (**upper** panels) and MART-1-specific CTLs (**lower** panels). (**B**,**C**) Quantification of CTL-mediated cytotoxicity. Graphs show the percentage of dead target cells among the CellTrace Violet-positive population from three independent experiments, each performed in duplicate, using CTLs derived from different healthy donors. Data are presented as mean ± SD. * *p* < 0.05, ** *p* < 0.01, *** *p* < 0.001.

We next asked whether antigen recognition and activation translated into direct target-cell killing. To this end, we performed a flow cytometry-based cytotoxicity assay using peptide-loaded T2 cells as target cells. T2 cells loaded with wild-type survivin LTLGEFLKL_96–104_ or MART1 EAAGIGILTV_26–35_ peptide were labeled with CellTrace Violet and co-cultured with the corresponding CTLs. Following co-culture, propidium iodide (PI) staining was used to identify dead cells within the labeled target-cell population (Figure 6A, representative examples). CTLs generated with m^1^Ψ-modified survivin or MART1 mRNA-transfected DCs efficiently killed T2 cells presenting their cognate peptide, whereas minimal killing was observed against T2 cells loaded with the control HIV-1 Pol peptide. Pre-incubation of T2 cells with the HLA class I-blocking antibody (W6/32 Ab) reduced the killing by approximately 50% ± 7% (*p* < 0.01), suggesting that the killing was class I restricted. Quantification of three independent experiments confirmed robust antigen-specific cytotoxicity for both survivin- and MART1-specific CTLs (Figure 6B,C, *p* < 0.001).

### 2.4. Antigen-Specific CTLs Recognize Endogenously Processed Antigens and Kill Tumor Cells in the Context of HLA-A2

Having established that m^1^Ψ-modified mRNA-transfected DCs generate CTLs capable of peptide-specific recognition, cytokine production, degranulation, and target-cell killing, we next asked whether these CTLs could recognize and eliminate tumor cells presenting endogenously processed antigen. To address this question, we used tumor cell lines expressing the investigated TAAs together with either compatible or incompatible HLA-A2 molecules, thereby providing a means to investigate antigen-specific recognition in the context of HLA-A2 restriction.

**Figure 7:**
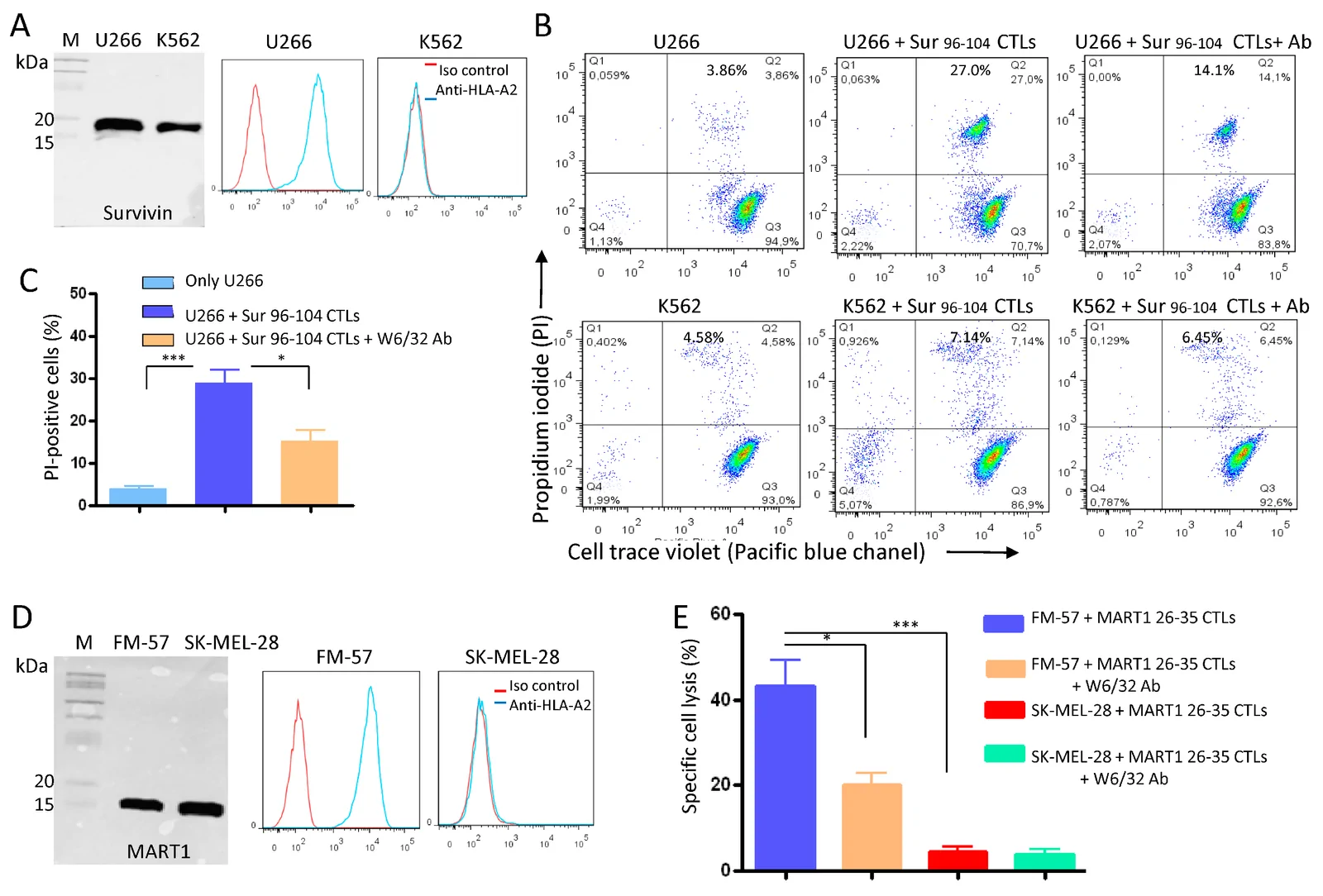
Antigen-specific CTL lines kill tumor cells expressing endogenous survivin or MART1. (**A**) Expression of survivin and HLA-A2 in tumor cell lines. Survivin protein expression in U266 (plasmacytoma) and K562 (erythroleukemia) cells was assessed by Western blotting using a survivin-specific monoclonal antibody. Surface HLA-A2 expression was analyzed by flow cytometry using an HLA-A2-specific monoclonal antibody (histograms). (**B**) HLA-A2-associate cytotoxicity of survivin-specific CTLs. U266 (HLA-A2^+^/survivin^+^) and K562 (HLA-A2^−^/survivin^+^) target cells (**upper** and **lower** panels, respectively) were labeled with CellTrace Violet and co-cultured with survivin-specific CTLs generated from HLA-A2^+^ healthy donors using autologous DCs transfected with m^1^Ψ-modified survivin mRNA. Target cells and CTLs were co-cultured at an effector-to-target ratio of 10:1 for 6 h. Following co-culture, cells were stained with propidium iodide (PI) and analyzed by flow cytometry. Representative dot plots are shown. (**C**) Quantification of tumor-cell killing by survivin-specific CTLs. Data represent results from three independent experiments, each performed in duplicate, using CTLs from different healthy donors and are presented as mean ± SD. (**D**) Expression of MART1 and HLA-A2 in melanoma cell lines. MART1 protein expression in FM-57 and SK-MEL-28 cells was assessed by Western blotting, and surface HLA-A2 expression was analyzed by flow cytometry using specific monoclonal antibodies (histograms). (**E**) HLA-A2-associated cytotoxicity of MART1-specific CTLs. FM-57 (HLA-A2^+^/MART-1^+^) and SK-MEL-28 (HLA-A2^−^/MART-1^+^) melanoma cells were used as target cells. The cells were co-cultured or not with MART1-specific CTLs generated from HLA-A2^+^ healthy donors using autologous DCs transfected with m^1^Ψ-modified MART1 mRNA at an effector-to-target ratio of 10:1 for 16 h. Following co-culture, target-cell viability was assessed using LDH release assay. Data represent results from four independent experiments, each performed in duplicate, using CTLs from different healthy donors and are presented as mean ± SD. * *p* < 0.05, *** *p* < 0.001.

We first evaluated survivin-specific CTL activity against the malignant hematopoietic cell lines U266 and K562. Western blot analysis confirmed substantial survivin expression in both cell lines, whereas flow cytometric analysis demonstrated HLA-A2 expression in U266 cells but not in K562 cells (Figure 7A, histograms). This combination provided an informative experimental system in which both target cells expressed the antigen, but only U266 cells expressed the restricting HLA allele. Next, we assessed tumor-cell killing using a flow cytometry-based cytotoxicity assay. U266 and K562 target cells were labeled with CellTrace Violet and co-cultured with survivin-specific CTLs at an effector-to-target ratio of 10:1 for 6 h, followed by propidium iodide (PI) staining to identify dead cells within the labeled target population (Figure 7B, representative example). A significant fraction of survivin^+^/HLA-A2^+^ U266 cells were efficiently eliminated by the survivin-specific CTLs (upper panels), whereas survivin^+^/HLA-A2^−^ K562 cells were largely resistant to killing (lower panels). Furthermore, blocking MHC class I-mediated recognition using W6/32 monoclonal (2 μg/mL) reduced CTL-mediated killing by approximately 49% ± 5% (*p* < 0.05), suggesting a class-I restricted killing (Figure 7B, right panel). Quantification of three independent experiments confirmed this HLA-A2- dependent cytotoxic response against U266 cells (Figure 7C).

We next asked whether the same principle applied to MART1-specific CTLs and melanoma cells. MART-1 expression and HLA-A2 surface expression were assessed in two melanoma cell lines, FM-57 and SK-MEL 28. As shown in Figure 7D, both cell lines expressed MART1 at high levels (Western blot), whereas only FM57 cells expressed HLA-A2 (histograms). The cytoxicity was assessed using the LDH release assay. MART1-specific CTLs generated following four weeks of repeated stimulation exhibited significant cytotoxicity against MART1^+^/HLA-A2^+^ FM57 cells, whereas little or no killing was observed against MART1^+^/HLA-A2^−^ SK-MEL 28 cells (Figure 7E). Consistent with the survivin experiments, blockade of HLA-A2 with the W6/32 antibody (2 μg/mL) reduced CTL-mediated killing of FM-57 cells by approximately 40% ± 6% (*p* < 0.05). CTLs generated in this study are not merely capable of recognizing exogenously loaded peptide; they can recognize endogenously processed survivin and MART1 and translate this recognition into effective tumor-cell killing. Although the data support antigen-specific killing, difference in the intrinsic sensitivity of the cell lines to cytotoxicity and a potential contribution of alloreactive cytotoxicity cannot be excluded.

### 2.5. Effect of UTRs and poly(A) Tails on Survivin and MART-1 mRNA Translation

**Figure 8:**
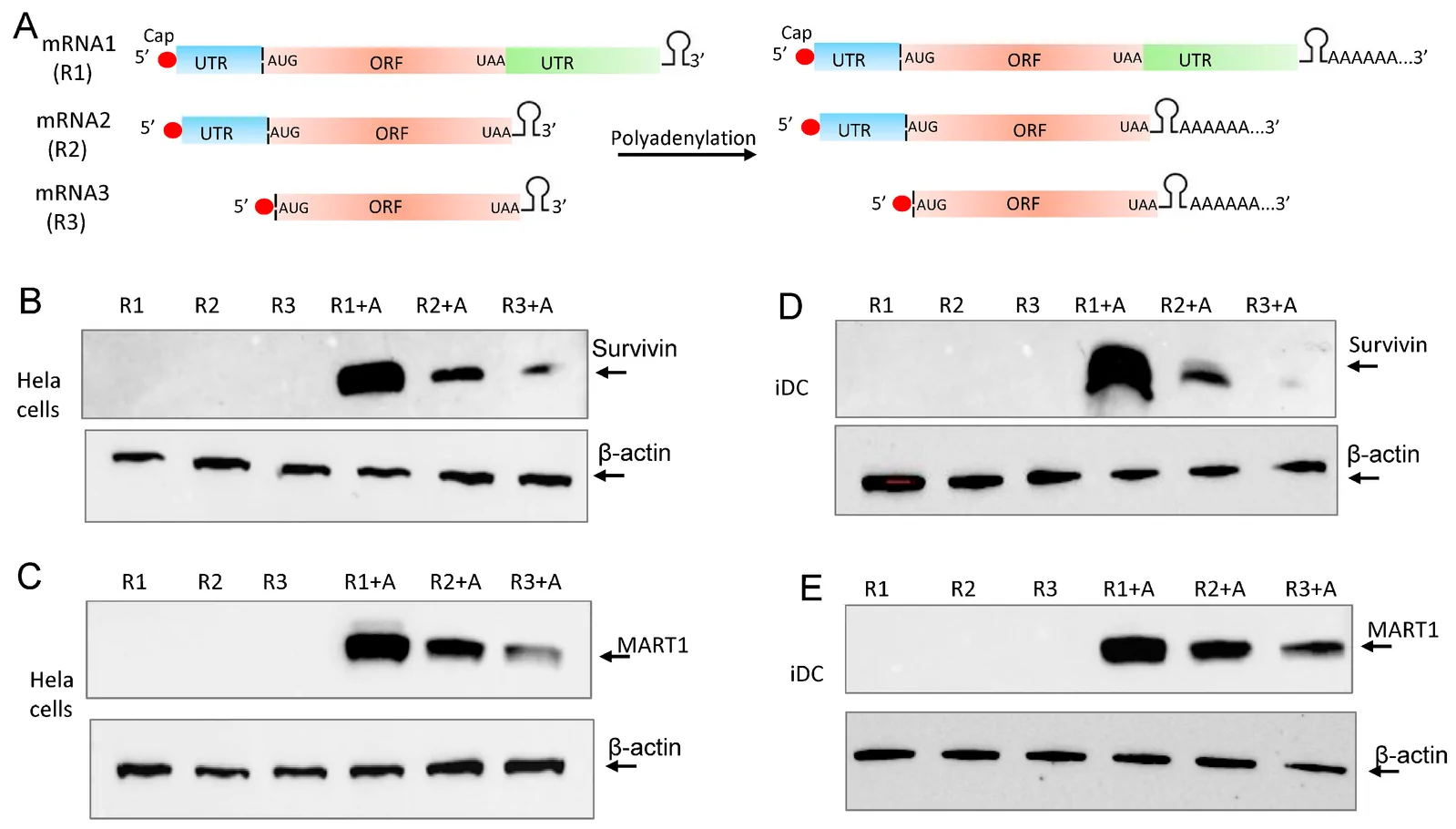
Effect of mRNA structural elements on translation. (**A**) Schematic representation of the different mRNA constructs tested. Each m1ψ-modified mRNA variant was transcribed without poly(A) tail, followed by polyadenylation after mRNA purification. HeLa (panels (**B**,**C**)) or iDC (panels (**D**,**E**)) cells were transfected with 750 ng dose of each non-polyadenylated mRNA variant: (1) mRNA 1 (R1) containing both 5′ and 3′ UTRs, (2) mRNA2 (R2) containing only the 5′ UTR, and (3) mRNA3 (R3) containing only the Kozak sequence. In parallel, cells were transfected with the corresponding polyadenylated mRNAs poly(A) + (R1 + A, R2 + A, R3 + A). After 18 h, protein expression was analyzed by Western blotting using either survivin- (Panels (**B**,**D**)) or MART1-specific monoclonal antibody (Panels (**C**,**E**)). The upper section of each membrane was probed for the expression of β actin. The data are representative of at least 6 independent experiments. Signal quantification is presented in Figure S1.

To investigate the contribution of untranslated regions (UTRs) and polyadenylation to the expression of the survivin and MART1, three mRNA variants were generated for each antigen, differing in their structural organization. The transcripts contained either (i) both the 5′ and 3′ UTRs (mRNA1, R1), (ii) only the 5′ UTR (mRNA2, R2), or (iii) neither UTR (mRNA3, R3), with the coding sequence preceded by a Kozak consensus sequence at the 5′ end. A short stabilizing 3′ hairpin structure was included in all transcripts (Figure 8A and Table S1). Each of the three mRNAs was produced with m^1^ψ and with or without a poly(A) tail, allowing us to independently assess the contribution of UTRs and polyadenylation to antigen expression. mRNAs were initially IVT transcribed without a poly (A) tail, and polyadenylation was subsequently introduced enzymatically using poly(A) polymerase. None of the non-polyadenylated transcripts resulted in detectable translation in either HeLa cells (Figure 8B,C) or iDCs (Figure 8D,E), regardless of their UTR configuration. In contrast, the addition of a poly(A) tail (+A) led to antigen expression in all three transcript configurations (Figure 8B–E, right portion of the images). The highest expression was observed with transcripts containing both the 5′ and 3′ UTRs, followed by transcripts containing only the 5′ UTR, while transcripts containing only the Kozak sequence showed the lowest, but still detectable, expression. The same pattern was reproducibly found across 6 independent IVT preparations (Figure S1). A similar expression profile was also observed when the mRNA variants were tested at an equivalent molar dose (i.e., the same number of molecules per condition) (Figure S2A,B). Thus, under the experimental conditions used, differences in mRNA length did not substantially affect the level of protein expression. Overall, The data indicate that polyadenylation is important for the translation of survivin and MART1 mRNAs, whereas the presence of UTRs primarily enhances translation efficiency rather than being strictly required.

## 3. Discussion

Efficient generation of tumor-specific CTLs remains a major challenge in cancer immunotherapy and is particularly important for the development of adoptive T-cell therapy (ACT). Although naturally occurring antitumor T-cell responses can recognize malignant cells, they are often constrained by low precursor frequencies, immune tolerance, inadequate antigen presentation, and the immunosuppressive tumor microenvironment [46]. Ex vivo generation and expansion of tumor-specific CTLs provides an opportunity to overcome these limitations by producing defined populations of functional effector cells before therapeutic administration [47,48]. In the present study, we demonstrate that nucleoside modification of mRNA with m^1^Ψ substantially enhances antigen expression in human DCs and, importantly, translates this improvement into more efficient generation of functional tumor-specific CTLs.

A central observation of this study is the striking increase in antigen expression achieved with m^1^Ψ-modified mRNA in human DCs. Under otherwise identical transcription and transfection conditions, m^1^Ψ-modified mRNAs encoding survivin and MART1 produced substantially higher levels of antigen than their uridine-containing counterparts. This effect was observed not only in monocyte-derived iDCs but also in circulating conventional DC1 and DC2 subsets, extending the relevance of the finding to physiologically distinct human DC populations. The enhanced protein production was accompanied by markedly reduced induction of IFN-β and TNF-α following m^1^Ψ-mRNA transfection. These observations are consistent with previous studies showing that unmodified IVT mRNA can activate innate RNA-sensing pathways, including TLR7, TLR8, RIG-I, and PKR, which can interfere with translation and limit protein expression [19,20]. Importantly, our data demonstrate that this principle is particularly relevant in human DCs, where efficient antigen expression is essential for subsequent T-cell priming. Indeed, DCs transfected with m^1^Ψ-modified survivin mRNA generated substantially higher frequencies of survivin-specific CD8^+^ T cells recognizing two distinct HLA-A2-restricted survivin epitopes. The ability to expand responses against multiple epitopes is noteworthy because tumor heterogeneity and antigenic variation can limit the efficacy of responses directed toward a single epitope. A multi-epitope response may therefore provide broader tumor recognition and potentially reduce the likelihood of immune escape through loss or alteration of an individual epitope. Importantly, the same principle extended beyond survivin: m^1^Ψ-modified MART-1 mRNA enhanced antigen expression and promoted the expansion of MART1-specific CD8^+^ T cells. Overall, the data support an association between increased antigen expression following m1ψ modification and more efficient-specific CTL generation, rather than demonstrating a direct causal relationship. Indeed, we cannot exclude the possibility that differences in cytokine production, DC functional state, prolonged presentation of antigen, or other effects associated with m1ψ modification may also contribute to the observed difference in T-cell priming. Another possibility is that m^1^Ψ-associated ribosome stalling may activate the ribosome-associated quality control pathway, potentially enhancing MHC-I peptide presentation and subsequent CTL responses. 

The ability of the generated CTLs to recognize and kill tumor cells expressing endogenous antigen represents a critical advance beyond peptide–MHC dextramer staining or responses against artificially peptide-loaded T2 target cells. Indeed, survivin-specific CTLs efficiently eliminated HLA-A2^+^/survivin^+^ U266 cells but not HLA-A2-negative/survivin^+^ K562 cells, while blockade of HLA class I substantially reduced cytotoxicity. Similarly, MART1-specific CTLs efficiently killed HLA-A2^+^/MART1^+^ melanoma cells but showed little activity against HLA-A2-negative targets. These observations suggest that the generated CTLs recognize naturally processed tumor antigens through the canonical MHC class I pathway and can translate antigen recognition into tumor-cell killing.

Previous studies demonstrated that electroporation of DCs with approximately 10–20 µg of IVT mRNA can induce potent antigen-specific CTL responses [49,50,51,52,53]. While these studies established the feasibility of mRNA-loaded DCs for T-cell priming, electroporation represents an ex vivo cell-loading approach and is not directly applicable to lipid-based systemic mRNA delivery. In the present study, we used LipoFectMax -mediated transfection, a lipid-based approach that is more compatible with the principles of lipid nanoparticles-mediated mRNA delivery and achieved efficient antigen expression using only 0.5 µg mRNA per transfection. Moreover, we directly compared unmodified and m^1^Ψ-modified mRNAs under identical experimental conditions. This allowed us to distinguish the effect of nucleoside modifications from differences in transfection conditions or mRNA dose. Importantly, whereas previous studies primarily focused on the ability of mRNA-loaded DCs to induce antigen-specific CTLs, the present study directly evaluated antigen expression together with CTL induction. The observed association between enhanced antigen expression following m^1^Ψ modification and increased frequencies of functional antigen-specific CTLs therefore provides additional evidence that improved intracellular antigen production can contribute to more effective T-cell priming. This finding is potentially beneficial in the context of mRNA DC vaccines as well as the generation of CTLs for ACT. Notably, the ability of m1Ψ-modified mRNA-transfected DCs to generate functional CTLs against both antigens suggests that this platform can accommodate different classes of tumor antigens rather than being restricted to a particular antigenic or tumor context. Consistent with this notion, we were also able to generate Wilms’s tumor 1-(WT1)-specific CTLs using the same strategy [54]. WT1 is aberrantly expressed in multiple hematological malignancies and solid tumors and has been extensively investigated as a target for cancer immunotherapy [55,56].

The generation of tumor-specific CTLs from healthy-donor PBMCs may have particular translational relevance for adoptive immunotherapy. Healthy donors provide a readily accessible source of T cells that have not undergone prolonged exposure to the suppressive tumor microenvironment and may therefore retain substantial proliferative and functional potential. This approach could be particularly relevant in the post-hematopoietic cell transplantation setting, where donor-derived antigen-specific CTLs targeting leukemia-associated antigens such as WT1 or survivin could potentially augment the graft-versus-leukemia (GVL) effect while minimizing the transfer of broadly alloreactive T cells associated with graft-versus-host disease (GVHD) [57]. Rather than administering an unselected T-cell population, generation and expansion of defined antigen-specific CTLs may provide a means of enriching the therapeutic repertoire while reducing unwanted immune reactivity as suggested previously [58]. However, whether m^1^Ψ-generated CTLs can provide clinically meaningful antitumor activity in vivo compare with established ACT approaches, remain to be investigated.

With respect to mRNA structural requirements, our data do not challenge the widely held view that canonical untranslated regions and polyadenylation generally support efficient mRNA translation and immunological functionality [59]. However, they differ from those recently reported by Esprit et al., who demonstrated that antigen presentation could be achieved using simplified IVT mRNA constructs lacking canonical regulatory elements, including the poly(A) tail [60]. Because antigen presentation requires intracellular expression and processing of the encoded protein, these findings indicate that non-polyadenylated mRNA can, under certain experimental conditions, achieve sufficient antigen expression to support antigen presentation and activation of established T cell clones in vitro. In contrast, our results demonstrate a strict dependence on polyadenylation for detectable expression of survivin and MART1. None of the non-polyadenylated transcripts, irrespective of whether they contained both UTRs, only the 5′ UTR, or only a Kozak sequence, produced detectable protein expression in either HeLa cells or iDCs. Similarly, no expression was observed in HEK293T cells. Protein expression was assessed by Western blotting and no specific protein signal was detected for any of the non-polyadenylated variants, even after prolonged membrane exposure, indicating that protein expression was absent or below the detection limit of the assay. In contrast, enzymatic addition of a poly(A) tail resulted in readily detectable protein expression across all three transcript configurations. The apparent discrepancy with the findings of Esprit et al. is therefore unlikely to be attributable solely to differences in the biological endpoint, as antigen presentation itself requires intracellular production of the encoded TAA. Rather, the contrasting observations may reflect differences in the coding regions and/or overall mRNA architecture. It is also conceivable that specific transcript designs contain sequence or structural features that partially compensate for the absence of a poly(A) tail, whereas the survivin and MART-1 constructs investigated here do not. Further studies are required to better understand the basis for poly(A) tail non-requirement in the translation of certain TAA mRNAs.

In conclusion, the present study identifies nucleoside modification as an important determinant of mRNA performance in human DCs and establishes an association between enhanced antigen expression and the effective generation of functional tumor-specific CTLs. m1Ψ modification simultaneously attenuated innate inflammatory responses and enhanced antigen expression. In parallel, m1Ψ-modified mRNA promoted more efficient expansion of survivin- and MART1-specific CD8^+^ T cells with potent antigen-dependent effector functions and the capacity to recognize and kill tumor cell lines presenting endogenously processed antigen. The study further demonstrates that mRNA structural elements, particularly polyadenylation, can critically influence antigen expression and should therefore be empirically optimized for individual tumor-antigen constructs. Although further optimization is warranted, these results provide a mechanistic and functional rationale for incorporating m1Ψ-modified mRNA into DC-based cancer vaccines and ACT platforms.

## 4. Materials and Methods

### 4.1. Reagents

N1-methylpseudouridine and anti-reverse cap analog (ARCA) were purchased from TriLink Biotechnologies (San Diego, CA, USA). T7 RNA polymerase, RQ1 RNase-free DNase, ribonucleotides (rATP, rGTP, rUTP, and rCTP), and Antarctic phosphatase were purchased from Promega (Madison, WI, USA). Restriction enzymes and RNase inhibitor were obtained from New England Biolabs (Ipswich, MA, USA). LipoFectMax transfection reagent was purchased from ABP Biosciences (Rockville, MD, USA). Opti-MEM reduced-serum medium was obtained from Gibco (Thermo Fisher Scientific, Waltham, MA, USA), and X-VIVO 15 medium from Lonza (Basel, Switzerland). The CD141 (BDCA-3) MicroBead Kit and CD1c (BDCA-1) APC Kit were obtained from Miltenyi Biotec (Bergisch Gladbach, Germany). Cytokines, including IL-4, GM-CSF, IL-1β, TNF-α, IL-6, IL-7, IL-15, and IL-2, were purchased from R&D Systems (Minneapolis, MN, USA). Survivin, Mart-1, and HIV MHC dextramers were obtained from Immudex (Copenhagen, Denmark). Clarity Western ECL substrate was purchased from Bio-Rad (Hercules, CA, USA). Prostaglandin E2 and other chemicals were purchased from Sigma-Aldrich (St. Louis, MO, USA). The following monoclonal antibodies were used: anti-human survivin and anti-HLA class blocking antibody W6/32 (Santa Cruz Biotechnology, Dallas, TX, USA); PE-conjugated anti-human HLA-A2 and PE-conjugated anti-human CD107a (BD Pharmingen); APC-conjugated anti-human CD8 (Invitrogen); anti-CD14, anti-CD80, anti-CD86, and anti-HLA-DR (DakoCytomation, Glostrup, Denmark); anti-CD83 (BD Pharmingen, San Diego, CA, USA)); and anti-double-stranded RNA J2 (GenScript, Piscataway, NJ, USA).

### 4.2. Cells and Culture Conditions

The erythroleukemia K562, plasmacytoma U266, melanoma and SK-Mel28, and T2 cell lines were obtained from the American Type Culture Collection (ATCC, Manassas, VA, USA). FM-57 cell line was obtained from The European Searchable Tumor Line Database (ESTDAB). All cell lines were maintained in Dulbecco’s modified Eagle’s medium (DMEM) or RPMI supplemented with 10% fetal bovine serum and antibiotics.

Peripheral blood mononuclear cells (PBMCs) were isolated from buffy coats obtained from healthy adult HLA-A2-positive donors by density-gradient centrifugation using Lymphoprep (STEM Cell Technologies). According to the blood bank’s requirements, the use of these anonymized buffy coat samples does not require a separate Institutional Review Board approval or an additional Informed Consent Statement from the donors. A formal application to the blood bank is sufficient for obtaining the samples (our reference BIMRADA-Project F8). After cell isolation, the samples were assigned study identification numbers and cryopreserved until use.

Monocytes were isolated by plastic adherence as previously described [61]. Briefly, PBMCs (2 × 10^8^ cells/40 mL) were incubated in T-150 culture flasks for 2 h at 37 °C in a humidified incubator. Following gentle washing with pre-warmed medium, a fraction of the adherent cells were cultured in X-VIVO 15 medium supplemented with recombinant human IL-4 (50 ng/mL) and GM-CSF (50 ng/mL) for 5 days to generate immature dendritic cells (iDCs). The remainder of the freshly isolated monocytes were cryopreserved until use. The non-adherent, T-cell-enriched fraction obtained after monocyte isolation was used to isolate CD8+ T cells using magnetic bead-conjugated anti-CD8 antibodies (Invitrogen Dynal AS, Oslo, Norway). Purified CD8+ T cells were washed and cryopreserved until use. Conventional circulating dendritic cell subsets, CD141+ (cDC1) and CD1c+ (cDC2), were isolated from PBMCs using magnetic-activated cell sorting (MACS). Briefly, PBMCs were labeled with the corresponding antibody-conjugated magnetic beads and purified using an autoMACS Pro Separator according to the manufacturer’s instructions (Miltenyi Biotec). Purified cells were cryopreserved until use.

### 4.3. Cloning and Plasmid DNA Preparation

Synthetic genes encoding the optimized open reading frames of survivin (accession no. NM_001168.3) and Mart-1 (MART-1/Melan-A, accession no, NM_005511.2) were synthesized by GenScript and cloned into the pCIpA102 plasmid harboring the T7 promotor for in vitro transcription, as previously described [8,62]. The coding sequences were flanked by 5′ and 3′ β-globin untranslated regions (UTRs). Plasmid DNA was isolated from overnight bacterial cultures using the NucleoSpin Plasmid Isolation Kit (Macherey-Nagel, Düren, Germany). The plasmids were linearized with MfeI and verified by electrophoresis on 1% agarose gels. Linearized DNA was purified by adding 1/10 volume of 3 M sodium acetate and 2.5 volumes of 100% ethanol, followed by incubation at −20 °C for 2 h and centrifugation at 14,000× *g* for 30 min at 4 °C. The DNA pellets were washed twice with 70% ethanol, air-dried, and resuspended in UltraPure nuclease-free sterile water. DNA concentration was determined by UV/Vis spectroscopy using a NanoDrop 1000 spectrophotometer, and purified DNA was stored at −20 °C until use.

To determine the structural elements required for effective survivin and MART1 mRNA translation, optimized open reading frames containing either (i) both the 5′ and 3′ UTRs, (ii) only the 5′ UTR, or (iii) neither UTR were designed and cloned into BamHI/EcoRI-cleaved pUC57 (GenScript). In variants lacking the 5′ UTR, the coding sequence was preceded by a Kozak consensus sequence only. To increase the stability of mRNA, a short 3′ hairpin structure was added to all transcripts (Table S1). All sequences contained the canonical T7 promoter at their 5′ ends for in vitro transcription. The recombinant pUC57 plasmids were linearized using the EcoRI restriction enzyme, precipitated, and processed as described above before being used as templates for in vitro transcription.

### 4.4. In Vitro Transcription

mRNAs encoding human survivin or MART-1 TAA were synthesized by in vitro transcription (IVT) using T7 RNA polymerase from MfeI-linearized plasmid DNA templates (1 μg per 100 μL reaction). All ribonucleotides were used at a final concentration of 5 mM, except GTP, which was reduced to 2 mM to improve capping efficiency. Anti-reverse cap analog (ARCA; 8 mM) was included in the transcription reaction. To minimize inter-sample variation, all reagents were included in the master mix except m^1^ψ and uridine. The mRNA transcripts contained a 100-nucleotide poly(A) tail encoded in the DNA template [8]. IVT reactions were incubated at 37 °C for 3 h, followed by treatment with RQ1 RNase-free DNase I (1 U/μg DNA template) for 10 min at 37 °C to remove residual DNA. Residual double-stranded RNA (dsRNA) was first removed using Eppendorf cellulose columns, as described by Baiersdörfer et al. [39]. The single-stranded mRNA recovered in the early flow-through was precipitated by LiCl-based precipitation and further purified using the MEGAclear kit according to the manufacturer’s instructions (Thermo Fisher Scientific, Waltham, MA, USA). RNA concentration and purity were assessed by spectrophotometry, and RNA integrity was evaluated by agarose gel electrophoresis. The A260/A280 ratio was around 2.0. Purified mRNAs were diluted to the required concentration, aliquoted into RNase-free tubes, and stored at −80 °C until use. Similarly, the various mRNA constructs cloned into pUC57 under the T7 promoter were IVT using m^1^Ψ and purified as described above. Following mRNA purification, an aliquot (40 µg) of each transcript was polyadenylated using E. coli poly(A) polymerase according to the manufacturer’s instructions (New England Biolabs), generating transcripts with poly(A) tails of approximately 120 nucleotides. The expression of these polyadenylated mRNAs was then compared with that of the corresponding non-polyadenylated transcripts in HeLa cells and iDCs.

### 4.5. Induction of Cytokines by the In Vitro Transcribed RNAs

Immature DCs were seeded in 48-well plates (Nunclon™ Delta Surface, Thermo Fisher Scientific, USA) at 3 × 10^5^ cells per 600 μL of X-VIVO 15 medium and transfected with IVT survivin mRNA using LipoFectMax. For each transfection, IVT mRNA (500 ng dose) was diluted in 100 μL Opti-MEM, while 4 μL LipoFectMax was separately diluted in 100 μL Opti-MEM. Both solutions were incubated at room temperature for 5 min, combined, and incubated for an additional 30 min at room temperature. The resulting complexes were added to the cells and incubated for 18 h at 37 °C in a humidified atmosphere containing 5% CO_2_. For each experiments, RNA samples were tested in duplicate. After incubation, culture supernatants were collected, and TNF-α and IFN-β levels were measured using the BD OptEIA™ Human TNF or IFN-β ELISA Set (BD Biosciences, USA). Absorbance was measured using a Sunrise microplate reader with Magellan™ software PRO v7.5 (TECAN, Switzerland).

### 4.6. Transfection of iDCs

Monocytes from HLA-A2-positive donors were seeded in 6-well plates at 2 × 10^6^ cells per well in 3 mL X-VIVO 15 medium supplemented with IL-4 and GM-CSF (50 ng/mL each). On day 5, the medium was replaced with 2.5 mL fresh X-VIVO 15 medium containing IL-4 and GM-CSF. LipoFectMax (5 μL) was diluted in 250 μL Opti-MEM and incubated for 5 min at room temperature. In parallel, IVT mRNA (500 or 750 ng dose based on early studies [61]) was diluted in 250 μL Opti-MEM and incubated for 5 min. The two solutions were then combined gently and incubated for an additional 30 min at room temperature to allow complex formation. The resulting transfection mixture was added directly to the cells. Approximately 18 h later, protein expression was assessed by Western blotting. The same transfection conditions were used for the isolated blood cDC1 and cDC2. For maturation, mRNA-transfected iDCs were cultured for an additional 2 days in X-VIVO 15 medium supplemented with GM-CSF, IL-4, IL-6, IL-1β, and TNF-α (20 ng/mL each) and prostaglandin E2 (PGE2; 1 μg/mL). Flow cytometric analysis confirmed increased expression of CD80, CD83, CD86, and MHC class I and II molecules following maturation (Figure S3A,B). No significant differences were observed in the expression of surface maturation markers between mature uridine-mRNA transfected DCs and mature m1ψ-mRNA transfected DCs (Table S2). Mature DCs were subsequently used as antigen-presenting cells (APCs) to generate autologous CD8+ CTLs.

### 4.7. In Vitro Generation of CTLs

CD8^+^ T cells obtained during monocyte isolation were co-cultured with either irradiated (30 Gy) mature survivin- or MART1-transfected autologous DCs at a DC ratio of 1:5, based on preliminary optimization experiments. Cultures were maintained in X-VIVO 15 medium supplemented with interleukin-7 (IL-7; 5 ng/mL) and interleukin-15 (IL-15; 5 ng/mL). Low-dose interleukin-2 (IL-2; 10 IU/mL) was added 36 h after initial stimulation. Weekly re-stimulation was performed using freshly prepared, mRNA-transfected and matured DCs. Cultures underwent 3–4 stimulation cycles before downstream analyses.

### 4.8. MHC Dextramer Staining and Flow Cytometric Analysis

Survivin-specific CD8^+^ T cells were quantified using commercially available PE-conjugated peptide–MHC dextramers (HLA-A02:01) corresponding to the survivin epitopes LMLGEFLKL (anchor modified residues 96–104) and TLPPAWQPFL (residues 5–14). Mart-1-specific CD8^+^ T cells were quantified using PE-conjugated HLA-A02:01 MHC dextramers bearing the anchor-modified ELAGIGILTV peptide. As a negative control, cells were stained with an HIV-pol specific MHC class I (HLA-A2) dextramer (ILKEPVHGV, residues 476–484). All dextramers were obtained from Immudex. Cells were resuspended in 50 μL staining buffer (SB) (PBS, 1% BSA, 3mM ETDA) and the staining procedure was carried out by adding 5 μL of dextramers with incubation for 10 min in the dark at room temperature. Subsequently, APC-conjugated anti-CD8 monoclonal antibody was added (2.5 μL/sample) and cells were kept in the dark at 4 °C for 30 min. After two washing steps, cells were resuspended in 300 μL SB and analyzed on a BD FACSCanto II flow cytometer (BD Biosciences, San Jose, CA, USA). Lymphocytes were first gated based on their forward and side scatter (FSC/SSC) properties, and cell aggregates were excluded by doublet discrimination using FSC-A versus FSC-H. Subsequently, CD8^+^ T cells were identified by gating on the CD8^+^ population using APC-conjugated anti-CD8, and dextramer-positive cells were identified based on PE fluorescence (dextramer^+^). PE-and APC single-stained controls were used to establish the compensation settings for flow cytometric analysis. Data were analyzed using FlowJo software and dextramer-positive CD8^+^ T cells were quantified and expressed as a percentage of total CD8^+^ T cells.

### 4.9. Peptides and Loading of T2 Cells

Wild-type/native survivin LTLGEFLKL_96–104_, Mart-1 EAAGIGILTV_26–35_, and HIV Pol ILKEPVHGV_476–484_ peptides were purchased from GeneCust Europe (Dudelange, Luxembourg) at >90% purity, as verified by HPLC analysis. The HLA-A2-positive T2 cell line was pulsed with peptides for 2 h at a final concentration of 50 µg/mL in the presence of 5 µg/mL β2-microglobulin (BioVision). Following incubation, cells were washed three times to remove excess peptide and subsequently labeled with Cell Trace Violet and then they were used as targets in either a degranulation or a flow cytometry-based killing assay as described below. Of note, anchor-modified peptides were used for dextramer staining, wheras wild-type peptides were used for functional assays.

### 4.10. Degranulation/Surface Display of CD107a

T2 cells were loaded with wild-type survivin peptide LTLGEFLKL_96–104_ or Mart-1 peptide EAAGIGILTV_26–35_ and plated in X-VIVO 15 medium at a density of 2 × 10^4^ cells per well in round-bottom microtiter plates in triplicate wells. Survivin-specific CD8^+^ effector CTL line or MART1-specific CD8+ CTL line was added at effector-to-target (E:T = 10:1) ratios in a final volume of 200 µL per well. Plates were incubated for 6 h at 37 °C in a humidified atmosphere containing 5% CO_2_. During incubation, PE-conjugated anti-CD107a antibody (3 µL per well) and monensin (0.2 µL per well) were added to the cultures to assess degranulation and inhibit intracellular protein transport, respectively. Following incubation, cells were harvested, washed, and stained with APC-conjugated anti-CD8 antibody. Samples were then acquired using a BD FACSCanto II flow cytometer (BD Biosciences, San Jose, CA, USA). Data were analyzed using FlowJo software, with lymphocyte populations gated on their forward and side scatter (FSC/SSC) properties, and cell aggregates were excluded by doublet discrimination using FSC-A versus FSC-H. Subsequently, CD8^+^ T cells were identified by gating on the CD8^+^ population using APC-conjugated anti-CD8, and CD107a-positive population were identified based on PE fluorescence. PE-and APC single-stained controls were used to establish the compensation settings for flow cytometric analysis.

### 4.11. Flow Cytometry-Based Cytotoxicity Assay

A flow cytometry-based cytotoxicity assay was performed to assess target cell killing by survivin-specific CD8^+^ CTLs. Target cells (U266, K562) were labeled with Cell Trace Violet according to standard protocols, checked with flow cytometry and then seeded into a 96-well round-bottom plate. Tumor-antigen-specific CD8^+^ CTLs were added at an effector-to-target ratio of 10:1, as determined in preliminary experiments, and co-cultures were incubated for 6 h at 37 °C in a humidified atmosphere with 5% CO_2_. As a control, some target cells were preincubated with an MHC class I-blocking antibody (2 μg/mL) for 30 min at room temperature before the addition of effector T cells. Following incubation, cells were stained with propidium iodide (PI) to discriminate dead cells from viable violet-positive target cells. Samples were then analyzed by flow cytometry, and cytotoxicity was determined based on the proportion of violet-positive target cells that were PI-positive, indicating membrane compromise and cell death. The graphs show the original proportions of PI positive cells. Spontaneous target cell death is also shown. Similar conditions were used for peptide-loaded T2 cells as target.

### 4.12. Lactate Dehydrogenase (LDH) Release Assay

For melanoma cell lines (FM-57 and SK-ML-28), cell lysis was quantified using an LDH release assay according to the manufacturer’s instructions (Promega, Madison, WI, USA). Briefly, target cells were co-incubated with CD8^+^ CTLs generated as described above. Effector cells were added at an effector-to-target ratio of 10:1, as determined in preliminary experiments. Some target cells were preincubated with an MHC class I-blocking antibody (2 μg/mL) for 30 min at room temperature before the addition of effector T cells. Plates were incubated at 37 °C for 18 h, after which supernatants were collected, and LDH release was measured. As a positive control (maximum release), selected wells were treated with 5% Triton X-100. The percentage of specific cell lysis was calculated as follows: % Specific release = [(Experimental release − Background release)/(Maximum release − Background release)] × 100. Background LDH release was calculated as the sum of spontaneous LDH release from target cells and T cells cultured alone.

### 4.13. Western Blot Analysis

Western blot analysis was performed as previously described [62]. Briefly, transfected cells were harvested, washed with PBS, and lysed in buffer containing 1% NP-40 and protease inhibitors for 30 min on ice. Lysates were centrifuged at 16,000× *g* for 20 min at 4 °C, and protein concentrations were determined using the Bradford assay (Bio-Rad). Equal amounts of protein (30–40 μg) were separated by 12% SDS-PAGE and transferred to nitrocellulose membranes. Membranes were blocked with 5% non-fat milk in TBS-T buffer (Tris-buffered saline with Tween-20) and incubated overnight at 4 °C with the appropriate primary antibodies. After washing, membranes were incubated with HRP-conjugated secondary antibodies, and protein bands were detected using Clarity Western ECL substrate (Bio-Rad) and visualized with a ChemiDoc™ MP Imaging System. β-Actin was used as the loading control and detected using an anti-β-actin monoclonal antibody. Densitometric analysis was performed using Image Lab™ software v6.10, and target protein expression was normalized to β-actin signals.

### 4.14. Statistical Analysis

The data of independent experiments were summarized and displayed as mean plus/minus standard deviation. The difference between control and treated samples was analyzed using Student’s *t*-test. One-way analysis of variance (ANOVA) was used for multiple comparisons. For comparisons of CTL generation using the mRNA variants and cells from the same donors, the paired nature of the data was taken into account by using paired statistical tests, with each donor serving as a matched pair. For two-group comparisons, a paired Student’s *t*-test was used. Statistical analysis was carried out using GraphPad Prism 4.0 software. The value of *p* < 0.05 was considered statistically significant.

## Data Availability

The original contributions presented in this study are included in the article/Supplementary Material. Further inquiries can be directed to the corresponding author.

## Supplementary Materials

The following supporting information can be downloaded at: https://www.mdpi.com/article/10.3390/ijms27209191/s1.

## Abbreviations

ACT	Adoptive Cell Therapy
BDCA-1	Blood Dendritic Cell Antigen-1
BDCA-3	Blood Dendritic Cell Antigen-3
cDC	Conventional Dendritic Cell
cDC1	Conventional Dendritic Cell type 1
cDC2	Conventional Dendritic Cell type 2
CD1c	Cluster of Differentiation 1c
CD107a	Cluster of Differentiation 107a
LAMP-1	Lysosomal-Associated Membrane Protein 1
CD141	Cluster of Differentiation 141 (Thrombomodulin)
CTL	Cytotoxic T Lymphocyte
DC	Dendritic Cell
dsRNA	Double-Stranded RNA
GVL	Graft-Versus-Leukemia
GVHD	Graft-Versus-Host Disease
HLA	Human Leukocyte Antigen
HLA-A2	Human Leukocyte Antigen-A02 (HLA-A0201)
IFN-β	Interferon-Beta
IFN-γ	Interferon-Gamma
iDC	Immature Dendritic Cell
IVT	In Vitro Transcription
J2	J2 monoclonal antibody specific for double-stranded RNA
Kozak	Kozak consensus sequence (translation-initiation sequence)
LAMP-1	Lysosomal-Associated Membrane Protein 1
LDH	Lactate Dehydrogenase
m1Ψ	N1-Methylpseudouridine
MART1	Melanoma Antigen Recognized by T Cells 1
MHC	Major Histocompatibility Complex
PBMC	Peripheral Blood Mononuclear Cell
PI	Propidium Iodide
PKR	Protein Kinase R (double-stranded RNA-activated protein kinase)
RNA	Ribonucleic Acid
TAA	Tumor-Associated Antigen
TLR	Toll-Like Receptor
TNF-α	Tumor Necrosis Factor-Alpha
UTR	Untranslated Region
WT1	Wilms Tumor 1
