# Supplementary material for: N1-Methylpseudouridine-Modified mRNA-Transfected Dendritic Cells Enable Efficient Generation of Functional Tumor Antigen-Specific Cytotoxic T Lymphocytes
Source: Int J Mol Sci. 2026 Oct 16;27(20):9191. doi: 10.3390/ijms27209191 (PMC13705057; doi:10.3390/ijms27209191)
Supplement: Supplementary file 1 [file ijms-27-09191-s001.zip › ijms-4581327-supplementary.pdf]

Supplementary Materials

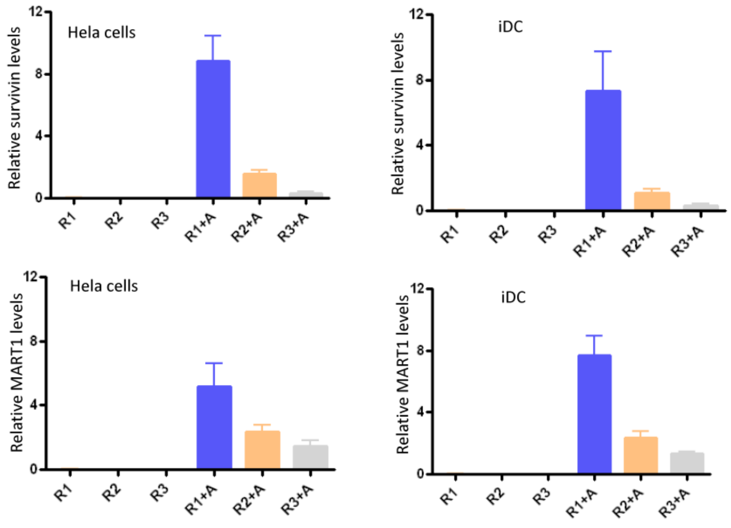

Figure S1. Effect of mRNA structural elements on translation. Survivin and MART1 expression was quantified by densitometry and normalized to β–actin for each sample (see Figure. 8). Values are presented as mean ±SD of 6 independent experiments.

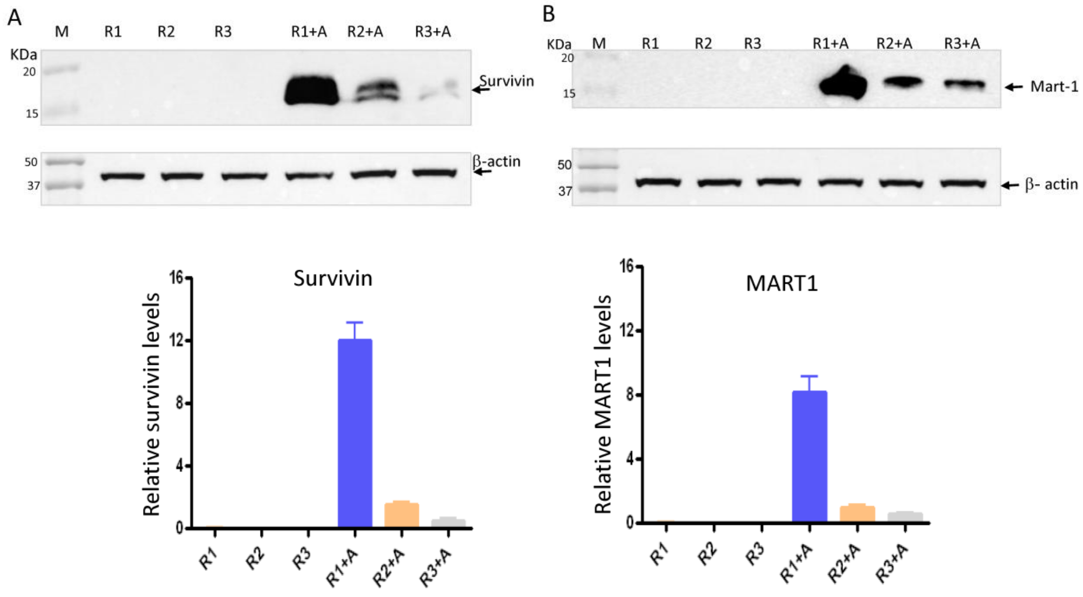

Figure S2A and B. Effect of mRNA structural elements on translation. Hela cells were transfected with mRNA 1 (R1) containing both 5′ and 3′ UTRs, (2) mRNA2 (R2) containing only the 5′ UTR, and (3) mRNA3 (R3) containing only the Kozak sequence. In parallel, cells were transfected with the corresponding polyadenylated mRNAs poly(A)+ (R1+A, R2+A, R3+A). All mRNA variant were tested at 1nM. After 18 h transfection time, protein expression was analyzed by Western blotting using either survivin- (Panel A) or MART1-specific monoclonal antibody (Panel B). The upper section of each membrane was probed for the expression of β–actin. Lower panels. The immunoreactive signals were quantified by densitometry and normalized to β-actin for each sample. Values are presented as mean ± SD of two independent experiments.

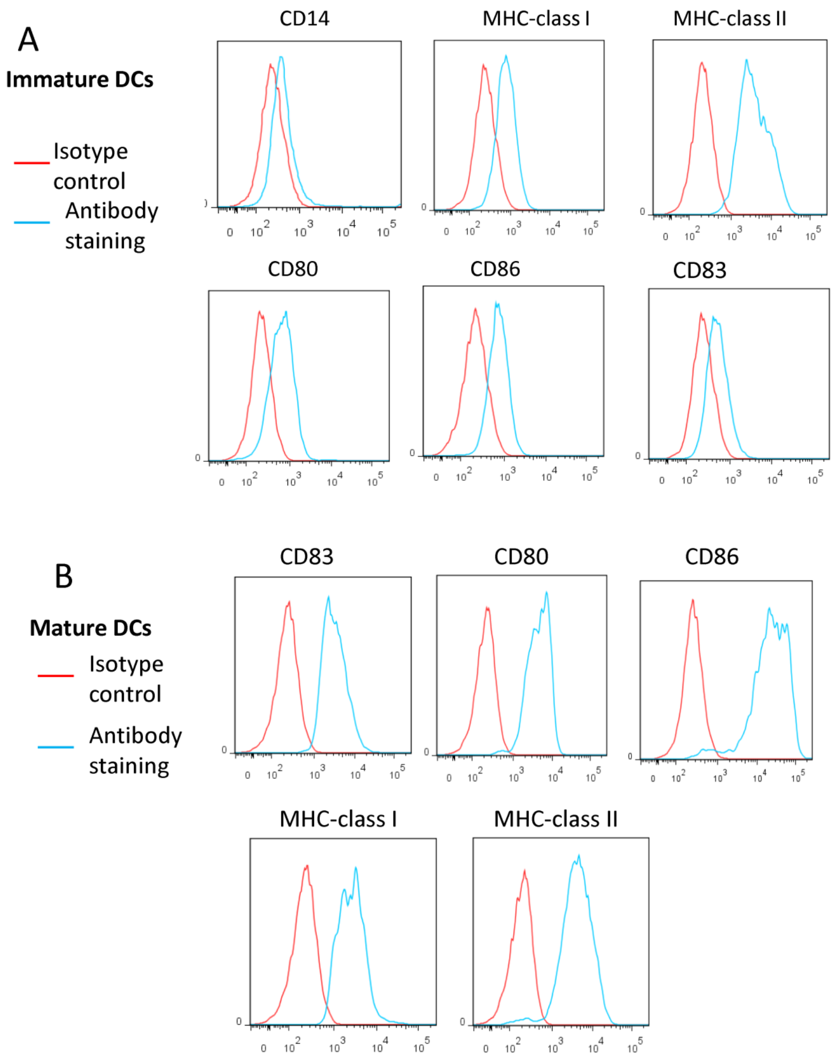

Figure S3A and B. Representative analysis of activation markers expressed by immature (**A**) and mature (**B**) dendritic cells (DCs) used for the generation of cytotoxic T lymphocytes (CTLs), as assessed by flow cytometry using specific monoclonal antibodies. Mature DCs were transfected with uridine-survivin mRNA.

Table S1: Survivin and MART1 mRNA sequences used in this study. The start codon (Met) is indicated in red, and the added 3' hairpin structure is underlined

Survivin mRNA variants

mRNA1

5'gacatttgcttctgacacaactgtgtgccaccatgggcgccccgacactgccgccagcctggcagcccttcctgaaggaccacagaatctccaccttcaagaac
tggcccttcctggaaggctgcgcctgcaccccccgagagaatggccgaggccggcttcatccactgccccaccgagaacgagcccgacctggcccaatgtttcttct
gcttcaaggagctggaaggctgggagcccgacgacgaccccatcgaggaacacaagaagcacagcagcggatgcgccttcctgagcgtgaagaagcagttcg
aggaactgaccctgggcgagttcctgaagctggacagagagcgggccaagaacaagatcgccaaagagacaaacaacaagaagaaggagttcgaggaaac
cgccaagaaagtgcggagagccatcgagcagctggctgccatggactgagctcgctttcttgctgtccaatttctattaaaggttcctttgttccctaagtccaacta
ctaaactgggggatattatgaagggccttgagcatctggattctgcctccaaaggctcttttcagagccaccca-3'

mRNA2

5'gacatttgcttctgacacaactgtgtgccaccatgggcgccccgacactgccgccagcctggcagcccttcctgaaggaccacagaatctccaccttcaagaac
tggcccttcctggaaggctgcgcctgcaccccccgagagaatggccgaggccggcttcatccactgccccaccgagaacgagcccgacctggcccaatgtttcttct
gcttcaaggagctggaaggctgggagcccgacgacgaccccatcgaggaacacaagaagcacagcagcggatgcgccttcctgagcgtgaagaagcagttcg
aggaactgaccctgggcgagttcctgaagctggacagagagcgggccaagaacaagatcgccaaagagacaaacaacaagaagaaggagttcgaggaaac
cgccaagaaagtgcggagagccatcgagcagctggctgccatggactgaaaccaaaggctcttttcagagccaccca-3'

mRNA3

5’gccaccatgggcgccccgacactgccgccagcctggcagcccttcctgaaggaccacagaatctccaccttcaagaactggcccttcctggaaggctgcgcct
gcaccccccgagagaatggccgaggccggcttcatccactgccccaccgagaacgagcccgacctggcccaatgtttcttctgcttcaaggagctggaaggctgg
gagcccgacgacgaccccatcgaggaacacaagaagcacagcagcggatgcgccttcctgagcgtgaagaagcagttcgaggaactgaccctgggcgagttcc
tgaagctggacagagagcgggccaagaacaagatcgccaaagagacaaacaacaagaagaaggagttcgaggaaaccgccaagaaagtgcggagagccat
cgagcagctggctgccatggactgaaaccaaaggctcttttcagagccaccca-3'

MART1 mRNA variants

mRNA1

5'gacatttgcttctgacacaactgtgtgctagcgccaccatgcctagagaagatgcccacttcatctatggctaccccaagaaaggacatggccacagctacacc
accgccgaggaagctgctggcatcggtattctgaccgtgatcctgggcgtgctcctgctgatcgggtgctggtactgccgccgccggaacggctacagagccctg
atggacaagtccctgcacgtgggcacacagtgtgcattgactcgccgctgtccacaggagggcttcgaccaccgggacagcaaggtgtccctccaagagaagaa
ctgcgagcctgtggtccccaatgcccctcctgcctacgagaagctgagcgccgagcagtctccaccgccctatagccccctaagaggcggccgcgctcgctttcttg
ctgtccaatttctattaaaggttcctttgttccctaagtccaactactaaactgggggatattatgaagggccttgagcatctggattctgcctccaaaggctcttttc
agagccaccca- 3'

mRNA2

5'gacatttgcttctgacacaactgtgtgctagcgccaccatgccaagagaagatgctcacttcatctatggttaccccaagaaggggcacggccactcttacacc
acggctgaagaggccgctgggatcggcatcctgacagtgatcctgggagtcttactgctcatcggctgttggtattgtagaagacgaaatggatacagagccttga
tggataaaagtcttcatgttggcactcaatgtgccttaacaagaagatgcccacaagaagggtttgatcatcgggacagcaaagtgtctcttcaagagaaaaact
gtgaacctgtggttcccaatgctccacctgcttatgagaaactctctgcagaacagtcaccaccaccttattcaccttaaccaaaggctcttttcagagccacccag
aattc-3'

mRNA3

5'gccaccatgccaagagaagatgctcacttcatctatggttaccccaagaaggggcacggccactcttacaccacggctgaagaggccgctgggatcggcatc
ctgacagtgatcctgggagtcttactgctcatcggctgttggtattgtagaagacgaaatggatacagagccttgatggataaaagtcttcatgttggcactcaatg
tgccttaacaagaagatgcccacaagaagggtttgatcatcgggacagcaaagtgtctcttcaagagaaaaactgtgaacctgtggttcccaatgctccacctgc
ttatgagaaactctctgcagaacagtcaccaccaccttattcaccttaaccaaaggctcttttcagagccacccagaattc-3'

Table S2: Expression of CD80, CD83 and CD86 surface markers by mature uridine mRNA- or m1ψ-mRNA-transfected dendritic cells

	Uridine mRNA-transfected DCs			m1ψ RNA-transfected DC		
Markers	CD80	CD83	CD86	CD80	CD83	CD86
MFI	2639 (±500)	2299 (±451)	3755 (±745)	2450 (±372)	1857 (±255)	3322 (±652)

Immature dendritic cells were transfected with either unmodified or modified survivin mRNA. After maturation, cells were harvested, and the expression of the CD80, CD83, and CD86 was analyzed by flow cytometry using specific antibodies. The mean fluorescence intensity (MFI) was determined for each marker. The data are represented as mean values ± SD from three independent DC preparations generated from three different donors.
